# Unit for women with schizophrenia in a community mental health service: Description of current and projected programs and quality evaluation measures

**DOI:** 10.1192/j.eurpsy.2023.2389

**Published:** 2023-07-19

**Authors:** A. González- Rodríguez, M. V. Seeman, M. Natividad, P. Barrio, E. Román, A. Balagué, J. P. Paolini, J. A. Monreal

**Affiliations:** 1 Mental Health, Mutua Terrassa University Hospital. University of Barcelona (UB). CIBERSAM, Terrassa, Spain; 2 Psychiatry, University of Toronto, Toronto, Canada; 3 Mental Health, Mutua Terrassa University Hospital. Fundació Docència i Recerca Mutua Terrassa. University of Barcelona (UB). CIBERSAM, Terrassa; 4 Addictions Unit. Psychiatry and Clinical Psychology., Hospital Clinic of Barcelona. University of Barcelona. IDIBAPS, Barcelona; 5 Mental Health, Mutua Terrassa University Hospital. Fundació Docència i Recerca Mutua Terrassa. University of Barcelona. CIBERSAM, Terrassa; 6 Mental Health, Mutua Terrassa University Hospital. Fundació Docència i Recerca Mutua Terrassa. University of Barcelona. CIBERSAM, Sant Cugat; 7 Mental Health, Parc Tauli University Hospital. Autonomous University of Barcelona (UAB). I3PT, Sabadell; 8 Mental Health, Mutua Terrassa University Hospital. Fundació Docència i Recerca Mutua Terrassa. University of Barcelona. CIBERSAM. Inst. Neurociències UAB, Terrassa, Spain

## Abstract

**Introduction:**

Women with schizophrenia require health interventions and safe spaces sufficiently different from those of men.

**Objectives:**

To describe units in two mental health outpatient services specialized in the treatment of women with schizophrenia and related disorders.

**Methods:**

Two units in Spain projected to treat women with schizophrenia and related disorders - Community Mental Health Programs (CMHU Rambla, CMHU Sant Cugat) will be described. Recruitment, assessment, intervention, and evaluation and satisfaction measures will be characterized, and the need to build-in safety precautions (policy/structural).

**Results:**

Demographics:(Preliminary 2021 data on the two services).

Patients attending (CMHU): 3,393. Forty-five per cent diagnosed with severe mental illness. Schizophrenia and related disorders: 873 patients. 58% women.

Staffing projected: 2 psychiatrists, 2 nurses, 1 clinical psychologist, 2 social workers.

Physical structure: Safe spaces for women/children.

Programs (offered currently and in planning stages): 1)Therapeutic Drug Monitoring/Adherence, 2)Individual/group patient/family sessions, 3)Perinatal Mental Health (preconception, pregnancy, lactation, postpartum, parent training/support), 4)Collaborative programs (primary care, medical specialties especially obstetrics/gynecology and endocrinology, trauma specialists, addiction experts), 5)prevention/intervention of suicide risk, 6)social services (single mothers, family issues, domestic abuse, sexual exploitation) 7)home-based services, 8)peer support, 9)physical activity, 10)psychoeducation for patients and families.

Planned quality evaluation measures: diagnostic assessment (reliability, long-term validity);regular treatment effectiveness evaluation (individualization of treatment plans, assessment of adverse effects of drugs, screening for metabolic syndrome/ physical health, family intervention, psychoeducation (individual/group) assessment of suicidal ideation and global functioning.

**Conclusions:**

Specific services for women with schizophrenia and related disorders represent an important resource to improve patient well-being and offer clinical care leading to individual recovery.

**Disclosure of Interest:**

None Declared

